# A comparative study of *k*-spectrum-based error correction methods for next-generation sequencing data analysis

**DOI:** 10.1186/s40246-016-0068-0

**Published:** 2016-07-25

**Authors:** Isaac Akogwu, Nan Wang, Chaoyang Zhang, Ping Gong

**Affiliations:** 1 School of Computing, University of Southern Mississippi, Hattiesburg, MS 39406 USA; 2 Environmental Laboratory, U.S. Army Engineer Research and Development Center, Vicksburg, MS 39180 USA

**Keywords:** Next-generation sequencing (NGS), *k*-mer, *k*-spectrum, Error correction, Sequence analysis, Bloom filter

## Abstract

**Background:**

Innumerable opportunities for new genomic research have been stimulated by advancement in high-throughput next-generation sequencing (NGS). However, the pitfall of NGS data abundance is the complication of distinction between true biological variants and sequence error alterations during downstream analysis. Many error correction methods have been developed to correct erroneous NGS reads before further analysis, but independent evaluation of the impact of such dataset features as read length, genome size, and coverage depth on their performance is lacking. This comparative study aims to investigate the strength and weakness as well as limitations of some newest *k*-spectrum-based methods and to provide recommendations for users in selecting suitable methods with respect to specific NGS datasets.

**Methods:**

Six *k*-spectrum-based methods, i.e., Reptile, Musket, Bless, Bloocoo, Lighter, and Trowel, were compared using six simulated sets of paired-end Illumina sequencing data. These NGS datasets varied in coverage depth (10× to 120×), read length (36 to 100 bp), and genome size (4.6 to 143 MB). Error Correction Evaluation Toolkit (ECET) was employed to derive a suite of metrics (i.e., true positives, false positive, false negative, recall, precision, gain, and F-score) for assessing the correction quality of each method.

**Results:**

Results from computational experiments indicate that Musket had the best overall performance across the spectra of examined variants reflected in the six datasets. The lowest accuracy of Musket (F-score = 0.81) occurred to a dataset with a medium read length (56 bp), a medium coverage (50×), and a small-sized genome (5.4 MB). The other five methods underperformed (F-score < 0.80) and/or failed to process one or more datasets.

**Conclusions:**

This study demonstrates that various factors such as coverage depth, read length, and genome size may influence performance of individual *k*-spectrum-based error correction methods. Thus, efforts have to be paid in choosing appropriate methods for error correction of specific NGS datasets. Based on our comparative study, we recommend Musket as the top choice because of its consistently superior performance across all six testing datasets. Further extensive studies are warranted to assess these methods using experimental datasets generated by NGS platforms (e.g., 454, SOLiD, and Ion Torrent) under more diversified parameter settings (*k*-mer values and edit distances) and to compare them against other non-*k*-spectrum-based classes of error correction methods.

## Background

Rapid generation and availability of massive amounts of DNA sequence data generated using next-generation sequencing (NGS) technologies at lower cost in comparison to traditional Sanger sequencing has led to a genuine ability to decipher genomes and perform ground-breaking biological research [1]. Some instances of the far reaching applications of NGS data include human genome profiling [2], microbiome research [3], de novo genome assembly [4], meta-genomics, and uncommon genetic variants identification [5]. In practice, NGS data has its challenges due to its relatively shorter read length and higher error rates in comparison to traditional Sanger sequencing [6], consequently constituting an undesirable property for downstream investigation.

The rates and types of sequencing error vary among different NGS technologies (Table 1) [7]. It is critically important to correct erroneous sequencing data before further downstream analysis. There exist multiple lines of evidence for negative effects of sequence errors on population-genetic studies [5] and output of alignment algorithms [8]. It has also been shown that correcting these errors has positive impact on downstream analysis such as improved genome assembly [4] and identification of single nucleotide polymorphism (SNP) [9].

**Table 1:** Sequence error rates for different NGS platforms in comparison with the traditional Sanger technology (updated as of 2012 at http://www.molecularecologist.com/next-gen-table-3c-2014/). See Glenn (2011) [41] for more details. Single-pass reads are those raw sequences that have not been subject to consensus adjustment incorporated in final base calling

Instrument	Primary error type	Single-pass error rate (%)	Final error rate (%)
Sanger—ABI 3730XL capillary (benchmark)	Substitution	0.1–1	0.1–1
454—all models	Indel	1	1
Illumina—all models	Substitution	~0.1	~0.1
Ion Torrent—all chips	Indel	~1	~1
SOLiD—5500XL	A-T bias	~5	≤0.1
Oxford Nanopore	Deletion	≥4	4
PacBio RS	Indel	~13	≤1

The general idea for correcting sequencing errors is that erroneous bases (i.e., nucleotides) in a DNA sequence read can be corrected using the majority of reads that have these bases correctly since errors occur infrequently and independently. Many error correction methods have been developed, some implemented as an integral part of a computational tool for de novo genome assembly [10, 11], while others as standalone tools [12–14]. Based on the type of data structure applied, these methods can be categorized into four main classes [15, 16]: *k*-mer spectrum-based (or *k*-spectrum-based) (e.g., Quake [17], Reptile [13] and Hammer [18]), suffix t-ree/array-based (HiTEC [19], SHREC [14] and Hybrid-SHREC [20]), multiple sequence alignment (MSA)-based (ECHO [12] and Coral [21]) and hidden Markov model (HMM)-based (PREMIER [22] and SEECER [23]).

Since Yang et al. [15] last reviewed error correction methods available as of December 2011, many new algorithms have been developed, especially in the fast-growing category of *k*-spectrum-based methods. In the past 2 years, at least five new *k*-spectrum-based methods have emerged, including Musket [24], Bless [16], Trowel [25], Lighter [26], and Bloocoo [27]. These new tools have made significant improvements in either memory usage, speed, or correction quality over previously existing tools. Authors of these new tools carried out a varying degree of comparison with other tools on synthetic and/or experimental datasets in order to demonstrate the superiority of their own tools. However, these authors chose different competitors for comparison and used different datasets and metrics for evaluation, making it hard to tell the relative strength and weakness between themselves.

The goal of this comparative study is to conduct an independent and unbiased assessment of the newly developed *k*-spectrum-based error correctors and to provide some guidance concerning how to choose a suitable error correction tool from the long list of existing methods. In particular, there is a lack of available studies that comprehensively investigate such factors as reference genome size, read length, and genome coverage depth that may differentially influence the performance of individual algorithm. Here, we first introduce the basics of *k*-spectrum error correction algorithms, and briefly review six sequence error correctors chosen for this study with an emphasis on distinctive features of each tool. Then, we present analytical results of method evaluation using simulated NGS datasets and make suggestions on which method is more suitable for a specific sequencing dataset. Finally, we discuss future perspectives and further studies required for assessing the performance of existing and newly developed error correction programs.

## Methods

### General framework of *k*-spectrum-based error correction methods

**Fig. 1:**
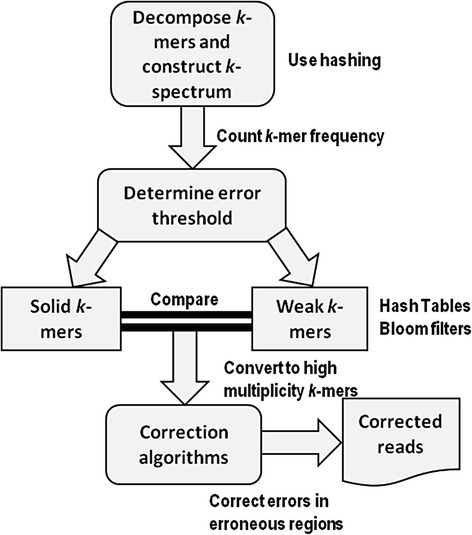
General framework of *k*-spectrum-based error correctors

Error correction methods based on *k*-spectrum originate from earlier implementation of de Bruijn graph assemblers using spectral alignment [28] and follow a generalized framework as shown in Fig. 1. A *k*-spectrum is the distribution of a set of decomposed distinct substring of length *k* (i.e., *k*-mer) observed in a group of reads. It counts the occurrence of all *k*-length contiguous strings represented as a vector within the spectrum feature space. The expectation is that errors in a sequence will result in a strong divergence at low *k*-mer frequencies compared to a sequence without errors. One challenge in error correction is that inconsistent genome sampling and genomic repeats may occur at high frequencies and consequently result in numerous equally susceptible correction possibilities. Owing to this, a frequently explored property of the *k*-mer spectra is the distribution composition of the spectra representing motif groups with varying sequence and bias frequencies [29]. This implies that based on their frequencies of occurrences, *k*-mers having small hamming distances are presumably of the same genomic locus and have to be corrected. *K*-spectrum-based correction starts by assigning a weighted value to each *k*-mer after extraction from sequencing reads. The value is assigned based on sorted count frequencies or base quality scores. By determining and selecting an acceptable error threshold [30, 31], *weak* (*insolid* or *untrusted*) *k*-mers with low frequencies are separated from *solid* (*trusted*) *k*-mers (with high frequencies). The reads with weak *k*-mers are considered for error correction by repeatedly converting them into solid *k*-mers until there are no more weak *k*-mers in the sequence. Hence, only solid *k*-mers will be kept after correction.

### Bloom filter

The majority of the methods investigated in this study apply Bloom filters as their data structure. As a space-efficient probabilistic data structure, a Bloom filter is used to test whether an element is a member of a set using binary array and multiple hash functions [32]. It can accurately determine a non-member element of the set. A query may return false positives but no false negatives, thus a bloom filter has a 100 % recall rate. A Bloom filter does not store the elements themselves but allows testing whether an object is certainly absent in the filter or has been probably added to the Bloom filter. For sequence error correction purposes, most methods implement the counting Bloom filter variant where array positions are not single bits but an *n*-bit counter. The efficiency of Bloom filters relies upon the number of bits in the array, the number of hash functions, and most importantly the quality of the hash functions.

### Selected *k*-spectrum-based error correction methods

Five recently published *k*-spectrum-based error correction methods, i.e., Musket [24], Bless [16], Bloocoo [27], Trowel [25], and Lighter [26], in addition to a relatively old program called Reptile [13] (published in 2010), were chosen for this comparative study. Reptile was chosen because it has been extensively evaluated and consistently proved to be an upper-scale performer in comparison with many other major error correction programs [13, 15, 16]. As summarized in Table 2, these methods differ greatly in error correction algorithms as well as in how hash tables and Bloom filters are implemented. The evaluated version is as follows: Reptile version 1.1, Musket version 1.1, Bless v0p23 for 64× Linux, Bloocoo 1.0.4-linux, Lighter version 1.1, and Trowel version 0.1.4.2.

**Table 2:** Characteristic features of the six *k*-spectrum-based methods investigated in the present comparative study which distinguish one method from others

Tools	Algorithm highlight	Data structure	Pros	Cons	Quality score	Target error type
Reptile	Explore multiple alternative *k*-mer decompositions and contextual information of neighboring *k*-mers for error correction	Hamming graph	Contextual information can help resolve errors without increasing *k* and lowering local coverage	Uses a single core (non-parallelized)	Used	Substitution Deletion Insertion
Musket	Multi-stage correction: two-sided conservative, one-sided aggressive and voting-based refinement	Bloom filter	Multi-threading based on a master–slave model results in high parallel scalability	A single static coverage cut-off to differentiate trusted *k*-mers from weak ones	Not used	Substitution
Bless	Count *k*-mer multiplicity; correct errors using Bloom filter; restore false positives	Bloom filter	High memory efficiency; handle genome repeats better; correct read ends	Cannot automatically determine the optimal *k* value	Not used	Substitution Deletion Insertion
Bloocoo	Parallelized multi-stage correction algorithm (similar to Musket)	Blocked Bloom filter	Faster and lower memory usage than Musket	Not extensively evaluated	Not used	Substitution
Trowel	Rely on quality values to identify solid *k*-mers; use two algorithms (DBG and SBE) for error correction	Hash table	Correct erroneous bases and boost base qualities	Only accept FASTQ files as input	Used	Substitution
Lighter	Random sub-fraction sampling; parallelized error correction	Pattern-blocked Bloom filter	No *k*-mer counting; near constant accuracy and memory usage	A user must specify *k*-mer length, genome length, and sub-sampling fraction α	Used	Substitution Deletion Insertion

Reptile [13] explores multiple alternative *k*-mer decompositions of an erroneous read and corrects errors by simultaneously examining Hamming (the number of positions at which two strings differ) distance-based correction possibilities for potentially erroneous *k*-mers using neighboring *k*-mers from the same read for correct contextual information. It also incorporates quality score information when available and has functionality to deal with ambiguous bases labeled as N’s. Reptile achieved a significant reduction in run time and memory usage and improvement in correction quality when compared with such existing methods as SHREC, Coral, Hybrid-SHREC, Quake, HiTEC, and ECHO [13, 15].

Musket [24] uses a multi-stage workflow including two-sided conservative correction, one-sided aggressive correction, and voting-based refinement. It computes the multiplicity of each *k*-mer in the hash table in order to filter out the stored unique *k*-mers using Bloom filter. A parallelized slave-master *k*-mer counting method is implemented to sort out unique *k*-mers and then generates *k*-mer coverage histograms to determine a cut-off for a *k*-mer spectrum for the coverage of likely correct and erroneous *k*-mers. The error correction stage initially uses a two-sided correction that conservatively corrects one substitution error, at most, in any *k*-mer of a read with the intention of finding a unique alternative base that makes all *k*-mers covering the position trusted. Significant improvement in speed can be achieved by evaluating only the leftmost and rightmost *k*-mers that cover the position. It then applies a one-sided correction to aggressively correct errors in the case of more than one error occurring in a single *k*-mer. Furthermore, to confine the number of false positives, error correction is conducted for each integer value from 1 to the maximal allowable number of corrections. The drawback is its reliance on alternative selection in the event that a *k*-mer is wrongly called to be trusted even though it contains sequencing errors or incorrect corrections. To overcome this drawback, look-ahead validation and voting-based refinement are implemented to assess the trustiness of a predefined maximal number (default = 2) of neighboring *k*-mers that cover the base position at which a sequencing error likely occurs. If all evaluated *k*-mers are trusted for a certain alternative on that position, this alternative is reserved as one potential correction.

Bless [16] uses a single minimum-sized Bloom filter and disk-based *k*-mer counting algorithm like disk streaming of *k*-mers (DSK) [33] and *k*-mer counter (KMC) [34] to achieve high memory efficiency for error correction, sequence repeat handling, and read end correction by read extension. Briefly, it counts *k*-mer multiplicity to sort out solid *k*-mers from weak *k*-mers, creates a *k*-mer multiplicity histogram to determine the multiplicity threshold *M*, and programs those solid *k*-mers into a Bloom filter. Weak *k*-mers are converted to their canonical forms using consecutive solid *k*-mers (known as *k*-mer islands) in their neighborhood or read end through Bloom filter querying. Bases in a weak *k*-mer that do not overlap with solid *k*-mers are modified. For instance, weak *k*-mers that exist between two consecutive solid *k*-mer islands *S*_1_ and *S*_2_ are corrected by using the rightmost *k*-mer of *S*_1_ and the leftmost *k*-mer of *S*_2_. Bless has three distinctive features: high memory efficiency, better handling of genome repeats, and more accurate error correction at read ends.

As part of the Genome Assembly & Analysis Took Box (GATB) [27], Bloocoo was developed to correct large datasets with low memory footprints by using DSK [33], a counting algorithm that requires a user to define a fixed amount of memory and disk space. Its error correction process is similar to CUSHAW [35], a procedure also used by Musket. In Bloocoo, the multi-set of all *k*-mers present in the reads is partitioned, and partitions are saved to disk. Then, each partition is separately loaded into memory in a temporary hash table. The *k*-mer counts are returned by traversing each hash table. Low-abundance *k*-mers are optionally filtered and solid *k*-mers are inserted in the Bloom filter based on a given threshold. With a multi-stage correction approach similar to Musket [24], correction is performed by scanning *k*-mers of a read, trying the other three different possible nucleotides at the error site, and checking if corresponding *k*-mers are in the set of solid *k*-mers. When several close errors occur, the pattern is more complex, and errors are corrected via a voting algorithm. Bloocoo distinguishes itself from other error correctors in the *k*-mer counting stage and the way that solid *k*-mers are stored in memory. By using only 11 bits of memory per solid *k*-mers, Bloocoo requires only 4-GB memory for the entire human genome re-sequencing read correction at 70× coverage.

Different from other tools, Lighter [26] samples *k*-mers randomly, i.e., sub-sampling fraction α rather than counting *k*-mers. It uses a pattern-blocked Bloom filter [36] to decrease the overall number of cache misses and improve memory efficiency. Lighter populates Bloom filter A with a *k*-mer subsample, followed by a simple test applied to each position of each read to compile a set of solid *k*-mers, and then stores the solid *k*-mers in Bloom filter B. A sequenced *k*-mer survives sub-sampling with probability of α, a user determined sub-sampling fraction that is set to be 0.10(70/C) with C being average coverage. For error correction, Lighter applies a greedy approach like that used in Bless [16] and extends a read when an error is located near the end of the read. Error correction is parallelized by using concurrent threads to handle subsets of the reads. Lighter maintains near constant accuracy and Bloom filter size as long as the sampling fraction is adjusted in inverse proportion to the coverage depth. However, a user has to specify *k*-mer length, genome length, and sub-sampling fraction α.

Trowel [25] is a highly parallelized and efficient error correction module for Illumina sequencing reads. The key difference to other tools is that Trowel relies on contiguity of high quality values instead of a *k*-mer coverage distribution to differentiate between solid and weak *k*-mers. The algorithm not only improves low quality bases but also iteratively expands the trusted *k*-mer set by including corrected *k*-mers. Trowel applies two different algorithms, Double Bricks & Gap (DBG) and Single Brick & Edges (SBE), to increase the likelihood that a correction can be made and to boost quality values. The *DBG* algorithm exploits an asymmetric *k*_1_-gap-*k*_2_ structure, where a gap is a single base, *k* = *k*_1_ + *k*_2_. The quality of the gap is boosted to the maximum quality value when the index relevant to gap-enclosing *bricks* contains the gap with high quality. The SBE algorithm is used because bases at read ends cannot be accessed by the *brick* index. Hence, a new edge-*k*-edge index is used to correct edges, where an edge is a single base, or increase their quality values as in the DBG algorithm.

### Dataset simulation

Reference genome sequences were downloaded from ftp://ftp.ncbi.nih/gov/genomes/refseq, including two bacteria genomes (*Escherichia coli* (EC) strain K-12 and *Bacillus cereus* (BC) strain ATCC 14579); and one invertebrate genome (*Drosophila melanogaster* (DM)). Synthetic, paired-end sequence read datasets were generated using ART with default Illumina profiles of empirical quality score distributions and error rates [37]. We chose to simulate Illumina-specific sequencing data because of the predominant status of Illumina sequencers among all NGS platforms. ART was used because it imitates the sequencing process with built-in, NGS platform-specific read error models and base quality value profiles parameterized empirically using large sequencing datasets [37]. All three types of errors (substitution, insertion, and deletion) for all major sequencing platforms are incorporated in simulated reads. The following default error rates were selected: 0.009 % and 0.015 % of insertion and 0.011 % and 0.023 % of deletion for the first and the second read, respectively. Base substitution is the dominant error type accounting for up to 98 % of all errors in Illumina sequencing data. The substitute rate in the simulated datasets varied, resulting in the overall error rate varying between 0.1 and 0.95 %, which is typical for Illumina sequencers (see Table 1). Details of the six simulated datasets are shown in Table 3 and these datasets can be downloaded at http://pinfish.cs.usm.edu/ngs_correction/.

**Table 3:** Synthetic paired-end Illumina sequencing datasets simulated using ART

Organism (dataset ID)	Accession number of reference genome assembly	ART simulation parameter				Genome size (MB)
		Read length (bp)	Genome coverage	Fragment/insert size	Error rate (%)	
*Escherichia coli* (EC-1)	GCF_000005845.2 (ASM584v2)	36	70×	200	0.866	4.6
*Escherichia coli* (EC-2)	GCF_000005845.2 (ASM584v2)	36	20×	200	0.866	4.6
*Escherichia coli* (EC-3)	GCF_000005845.2 (ASM584v2)	100	20×	200	0.952	4.6
*Bacillus cereus* (BC-1)	GCF_000007825.1 (ASM782v1)	56	50×	200	0.175	5.4
*Bacillus cereus* (BC-2)	GCF_000007825.1 (ASM782v1)	100	120×	300	0.109	5.4
*Drosophila melanogaster* (DM)	GCF_000001215.4 (Release 6)	100	10×	300	0.854	143

### Evaluation tools

**Fig. 2:**
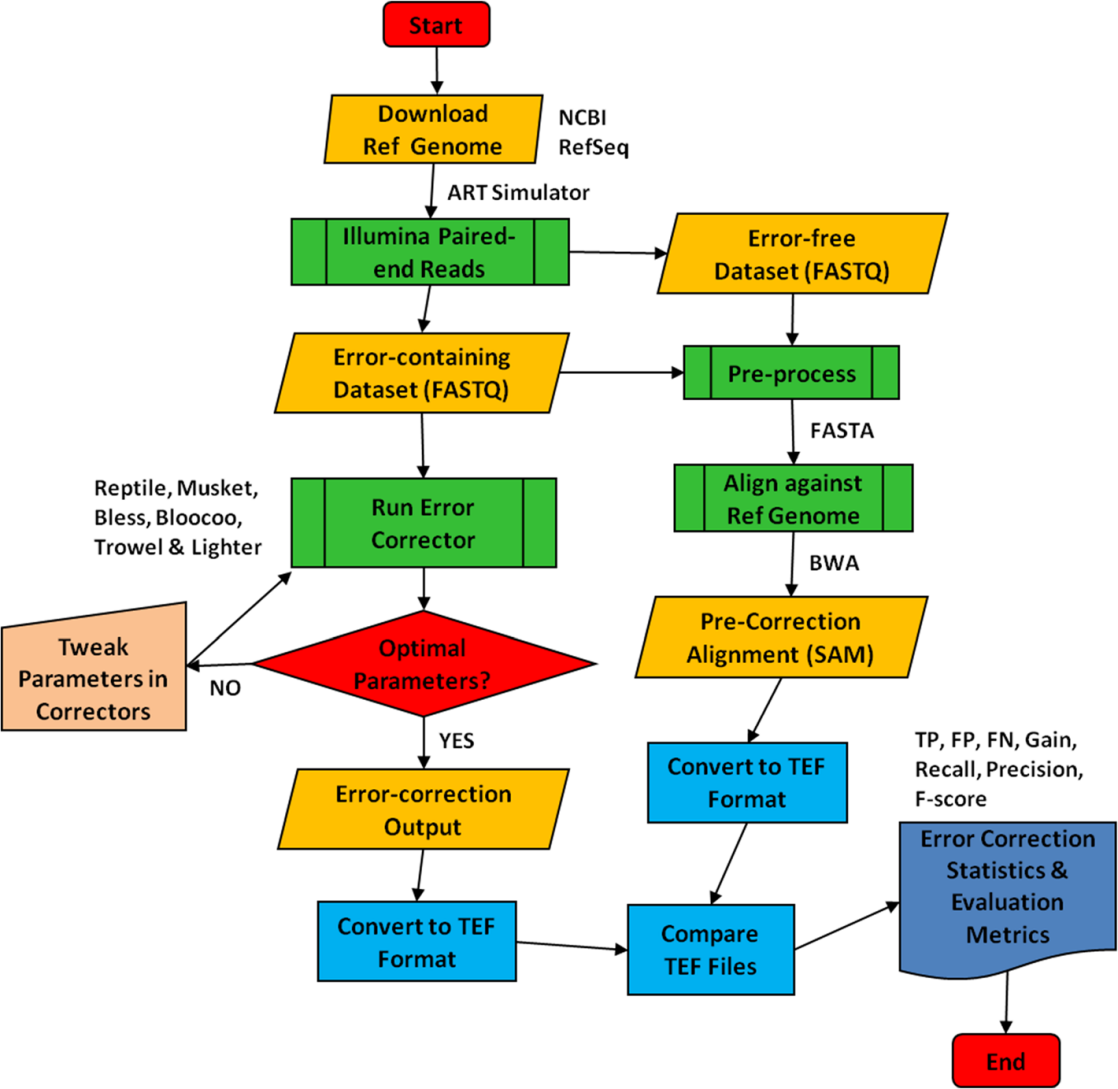
Workflow of error correction performance analysis using ECET (Error Correction Evaluation Toolkit [15]). See http://aluru-sun.ece.iastate.edu/doku.php?id=ecr for more information

Error Correction Evaluation Toolkit (ECET) version 1.1 [15] was used for performance analysis in order to take advantage of its neutral format known as target error format (TEF), which ensures that all methods are evaluated equally (Fig. 2). Furthermore, ECET produces error correction statistics and measures that can be directly used for performance evaluation. Burrows-Wheeler Aligner (BWA) version 0.6.1-r104 [38] was used for short read mapping with default settings except for the edit distance parameter set to 2 and 4 for short (36/56 bp) and long (100 bp) read length, respectively. Read alignment was performed so as to identify the difference between the reference genome and the erroneous sequence. SamToFastq, one of the Picard command line tools (Release 1.119, http://broadinstitute.github.io/picard), was used to validate the conversion of SAM (sequence alignment/mapping)-formatted files to FASTQ files [39] implemented by the sam-analysis.py script in ECET. KmerGenie version 1.6476 [40] was used to determine the optimal *k* to be selected for correction. DSK version 2.0.2-Linux [33] was used for counting *k*-mers for proper estimation of valid *k*-mers given an optimal *k*-mer length before error correction.

### Evaluation workflow and metrics

As shown in Fig. 2, the workflow consists of the following steps: sequencing dataset simulation, pre- and post-correction alignment to reference genome, parameter optimization for error correctors, and derivation of evaluation statistics and metrics (see http://aluru-sun.ece.iastate.edu/doku.php?id=ecr for more details). Briefly, both error-free and error-containing paired-end sequences were generated in FASTQ format by ART simulation. The error-free data served for the QA/QC purpose throughout the workflow. After converting FASTQ to FASTA (pre-process due to ECET’s header requirements before alignment), simulated sequences were aligned to a reference genome using BWA. The SAM alignment files produced by BWA were then converted to TEF format using ECET [15]. Error-containing datasets were corrected using error correction tools. The error correction outputs from these tools were converted to TEF files in ECET. The TEF files that generated pre- and post-correction were compared using the Comp2PCAlign script provided in ECET to produce statistics and metrics for performance assessment. Since the quality and accuracy of error correction tools are highly dependent on parameter (particularly *k*-mer) settings, we introduced an iterative optimization loop to select an optimal *k* value by implementing KmerGenie. In this loop, we also tweaked other parameters while maintaining the same optimal *k*-mer selected for a specific dataset. For instance, α is a user-defined parameter in Lighter and its default value is set by the formula 0.1(70/C), where C is the coverage depth.

We chose the following widely used metrics to evaluate correction quality [13, 15, 16, 24–27]: true positives (TP)—an erroneous base correctly changed to its true base; false positives (FP)—a true base incorrectly changed; false negative (FN)—incorrect base left unchanged; true negative (TN)—true base left unchanged; recall or sensitivity = TP/(TP + FN), precision = TP/(TP + FP), gain = (TP − FP)/(TP + FN), and F-score = 2 × ((precision × recall)/(precision + recall)).

### Operating systems

Computational experiments were conducted using multiple machines due to specific requirements of individual tools and varied sizes of synthetic testing datasets; hence, consideration was not given to the performance in terms of run time and memory usage but rather effectiveness and accuracy of read error correction. Due to requirement of Message Passing Interface (MPI), Bless was run on a Red Hat Enterprise Linux MPI cluster with 12 nodes, and each node had 12-GB memory and 8 cores running at a core speed of 2.93 GHz. For all other tools, datasets with a genome size >5 MB were run on a 64-bit Ubuntu 12.04 LTS Intel Core i7-3770 CPU@ 3.40 GHz machine with 8 cores and 8-GB memory. The *E. coli* datasets (genome size <5 MB) were run on a CentOS—64-bit Intel(R) Xeon(R) CPU E5630@ 2.53 GHz machine with 16 processors and a total memory of 296 GB.

## Results

The derived performance metrics are presented in Table 4. Bless and Bloocoo each failed to process one dataset, i.e., BC-2 and DM, respectively. A negative gain value means that more errors are introduced into the data than corrected. Five methods (Reptile, Bless, Bloocoo, Trowel, and Lighter) produced negative gains, mostly in processing EC-3. F-score is the most comprehensive measure of error correction performance. If setting F-score = 0.8 as the threshold for good performance, all methods except Musket underperformed with at least one dataset. Therefore, Musket was the best overall performer whereas Trowel was the worst one with five instances of underperformance.

**Table 4:** Performance analysis of six *k*-spectrum-based error correctors as evaluated using six synthetic Illumina datasets

Dataset	Method	TP	FP	FN	Recall	Gain	Precision	F-score
EC-1	Reptile	2335361	144751	451889	0.8378	0.7859	0.9416	0.8867
36 bp	Lighter	2695425	72843	91825	0.9671	0.9409	0.9737	0.9704
70×	Bless	2624659	48342	*56279*	*0.9790*	*0.9610*	0.9819	*0.9805*
*k* = 19	Bloocoo	2411701	*22259*	375549	0.8653	0.8573	*0.9908*	0.9238
	Musket	*2701885*	61096	85365	0.9694	0.9474	0.9779	0.9736
	Trowel	1246340	705438	1539825	0.4473	0.1941	0.6386	0.5261
EC-2	Reptile	681551	140039	114910	0.8557	0.6799	0.8296	0.8424
36 bp	Lighter	108241	58579	688220	0.1359	0.0624	0.6488	0.2247
20×	Bless	*779824*	18095	*16637*	*0.9791*	*0.9564*	0.9773	*0.9782*
*k* = 17	Bloocoo	689322	*6454*	107139	0.8655	0.8574	*0.9907*	0.9239
	Musket	767087	18182	29374	0.9631	0.9403	0.9768	0.9699
	Trowel	434885	19167	361576	0.5460	0.5220	0.9578	0.6955
EC-3	Reptile	105	461	876053	0.0001	-0.0004	0.1855	0.0002
100 bp	Lighter	858125	2446	18033	0.9794	0.9766	0.9972	0.9882
20×	Bless	746	872860	875412	0.0008	-0.9954	0.0009	0.0009
*k* = 24	Bloocoo	79790	3644539	796368	0.0911	-4.0686	0.0214	0.0347
	Musket	*873592*	*1645*	*2566*	*0.9971*	*0.9952*	*0.9981*	*0.9976*
	Trowel	155	178354	876003	0.0002	-0.2034	0.0009	0.0003
BC-1	Reptile	382043	22303	16602	0.9584	*0.9024*	0.9448	*0.9515*
56 bp	Lighter	331759	*15470*	*141618*	0.7008	0.6682	*0.9554*	0.8086
50×	Bless	*429017*	34018	11943	*0.9729*	0.8958	0.9265	0.9492
*k* = 27	Bloocoo	410156	24127	63221	0.8664	0.8155	0.9444	0.9038
	Musket	355015	47460	118362	0.7500	0.6497	0.8821	0.8107
	Trowel	55277	4976	26744	0.6739	0.6133	0.9174	0.7770
BC-2	Reptile	497425	116	208081	0.7051	0.7049	0.9998	0.8269
100 bp	Lighter	698089	159	7417	0.9895	0.9893	0.9998	0.9946
120×	Bless	–	–	–	–	–	–	–
*k* = 31	Bloocoo	27409	1278837	678097	0.0389	-1.7738	0.0210	0.0272
	Musket	*703882*	*68*	*1624*	*0.9977*	*0.9976*	*0.9999*	*0.9988*
	Trowel	652845	108	52661	0.9254	0.9252	0.9998	0.9612
DM	Reptile	*11702183*	187733	*517322*	*0.9577*	*0.9423*	0.9842	*0.9708*
100 bp	Lighter	42	23055867	12224293	0.0000	-1.8861	0.0000	0.0000
10×	Bless	11122683	*126388*	1101652	0.9099	0.8995	*0.9888*	0.9477
*k* = 21	Bloocoo	–	–	–	–	–	–	–
	Musket	11550483	163838	673852	0.9449	0.9315	0.9860	0.9650
	Trowel	1197127	384403	11027208	0.0979	0.0665	0.7569	0.1734

In the first column, dataset ID, read length, genome coverage, and the optimal *k* estimated using KmerGenie are shown. The values in TP, FP, and FN columns are numbers of bases. *Italicized values* denote the best performer with regard to a specific evaluation measure for a dataset. The symbol “–” indicates that a method failed to process a specific dataset

### Influence of read length on performance

**Fig. 3:**
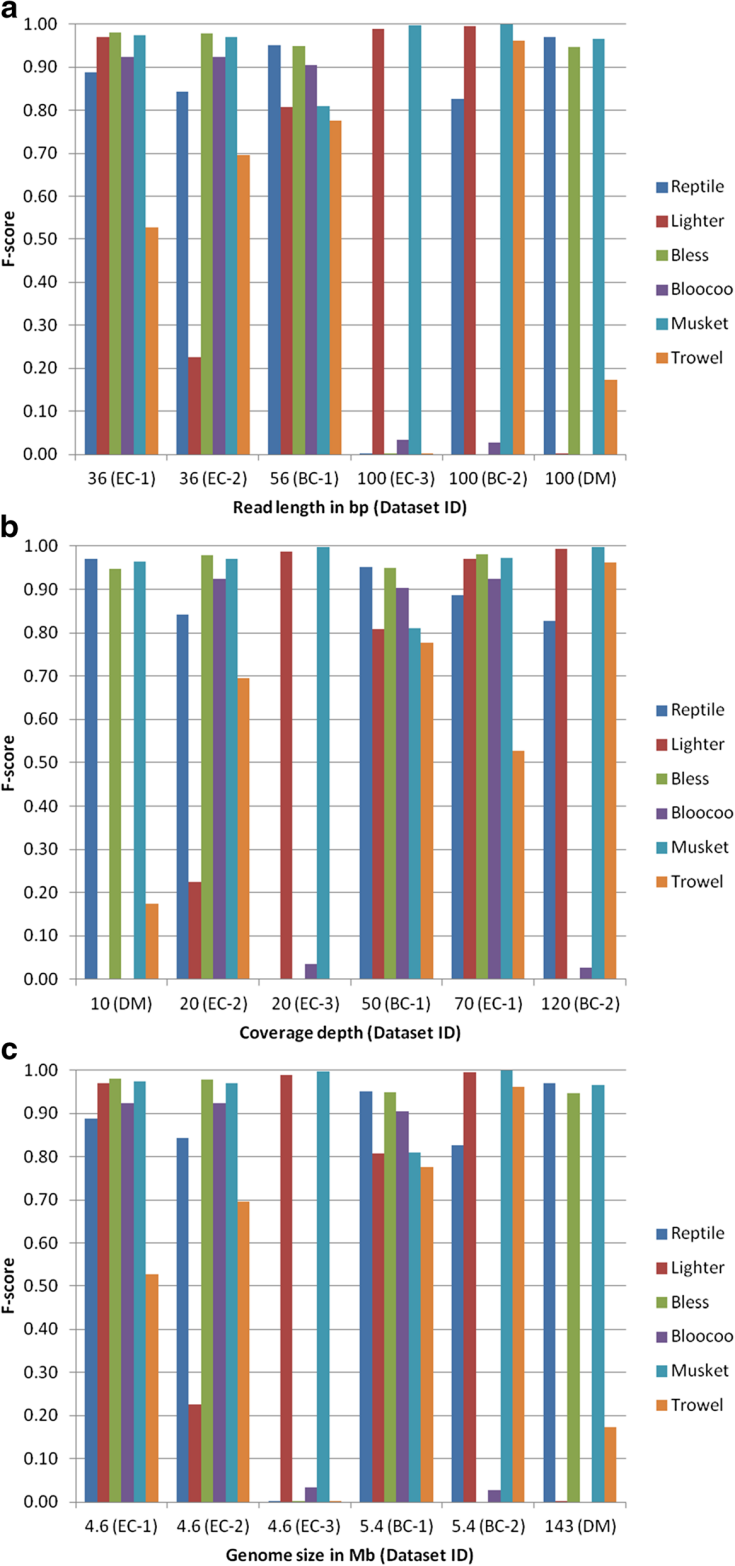
Impact of read length (**a**), coverage depth (**b**), and genome size (**c**) on the performance of six *k*-spectrum-based error correction methods. The six datasets are reordered according to the factor examined in order to show visually the effect of each factor on F-score for each method (see Table 3 for dataset, method, and F-score information)

Three datasets with a short read length of either 36 or 56 bp were processed by four to five methods to a satisfactory degree (F-score > 0.8, Fig. 3a). Only two, three, and four methods generated satisfactory results with the other three 100-bp datasets EC-3, DM, and BC-2, respectively. In general, read length has an adverse impact on tool performance, i.e., the longer the read length, the less superior a tool performs. This impact was the most pronounced on Bloocoo, which underperformed in all three long-read datasets. Musket was the most resistant tool because it performed well across all six datasets. For the other four tools, there appeared to exist interactive effects among read length, coverage depth and genome size because no clear-cut relationship between read length and performance was observable.

### Influence of genome coverage depth on performance

A medium coverage depth (50- and 70-fold) appeared to be preferred by all tested tools except Trowel (Fig. 3b). At a low depth (10- and 20-fold), Reptile and Bless performed well except for the long-read dataset EC-3. Lighter seemed to require a medium-to-high coverage depth (50-fold or higher). In case of low depth (20-fold), a longer read length might compensate for the loss of coverage depth, resulting in a satisfactory performance. At the highest coverage depth (120-fold), two tools failed (Bless) or underperformed (Bloocoo). Again, Musket showed the strongest resistance to variation in coverage depth.

### Influence of genome size on performance

Genome size is most likely a covariant that interacts with the other two factors (read length and coverage depth) because instances of underperformance occurred across the full spectrum of genome size (Fig. 3c). For small genomes (EC and BC), Musket was the best method, followed by Lighter and Reptile (both performed well in four of five datasets), then Bless and Bloocoo (three of five datasets), and Trowel ranked the last. For the large genome (DM), only three methods (Reptile, Bless and Musket) performed well.

## Discussion

Different Bloom filter variants were implemented in four of the six investigated methods to allow compression of the filter, storage of count data, and representation of maps in addition to sets [26] (also see Table 2). The other two methods (Reptile and Trowel) used hash tables, which do not yield false positive. Although Bloom filter’s space efficiency comes at the cost of false positives, all major error correction programs have reduced or minimized false positive rate by implementing various algorithms. Authors who developed these six tools had put lots of efforts in increasing speed and reducing memory footprint while maintaining or improving their correction quality. In the present study, we chose to focus solely on correction quality because speed and memory are no longer bottlenecking factors that limit the application of these tools.

Simulated datasets were used because correction accuracy could be directly measured. When real experimental datasets are used, only indirect evaluation metrics (e.g., N50 contig size and genome coverage of de novo assemblies and percentage of mapped reads in genome alignment) can be derived for performance assessment. We believe that the usage of real datasets in tool evaluation can provide insights that cannot be obtained from simulation studies. Nevertheless, extensive evaluations should be conducted using simulated datasets before moving on to real datasets. Authors of the six tools investigated in our study have performed evaluations using both synthetic and real datasets. In general, tools that perform well with synthetic datasets also work well with real datasets (see publications featuring Bless [16], Trowel [25], and Lighter [26]). There is a good correlation between performance metrics for simulated and real datasets.

Previous evaluations showed that Musket was consistently one of the top performing correctors for both simulated and real datasets when it was compared with several well-regarded programs: HiTEC, SHREC, Coral, Quake, Reptile, DecGPU, and SGA [24]. Here, we also demonstrated that Musket yielded better performance metrics than Reptile. When authors of Bless [16], Trowel [25], and Lighter [26] performed their comparative evaluations, they claimed that their own tools slightly outperformed Musket. However, if looking more specifically into simulated datasets, Musket performed equally well as the other three tools did (e.g., the synthetic 40× human chromosome 1 dataset used in [16]). Bloocoo shares a great deal of similarity with Musket, especially in the multi-stage error correction algorithm [24, 27]. They reportedly achieved similar correction accuracy as measured by recall and precision on a simulated dataset with 1 % error rate from human chromosome 1 at 70× coverage (see “Supplementary Material” in [27]). In the current study, these two programs did perform equally well on three datasets (EC-1, EC-2, and BC-1 with read length of 36 or 56 bp). However, Bloocoo underperformed or failed on the remaining three datasets with longer reads (100 bp), suggesting the existence of potential bottleneck factors in the scripts of Bloocoo that limit its application to longer reads.

An inherent difficulty in using any corrector is the challenge of choosing optimal parameters [13]. Very few tools have implemented automated choice of parameters sensitive to datasets being processed. Although Bless [16] can automatically choose an appropriate value for *M*, *k*-mer multiplicity threshold, it cannot select an optimal *k* and nor can other tools evaluated in this study (except for Reptile [13], which chooses *k* = log_4_|G|, where G is the genome length). We used KmerGenie [40] to determine an optimal *k* for each dataset. While it is possible that the *k* picked by KmerGenie may not be the optimal value for all six evaluated tools, we performed limited tests in tweaking *k* and other user-defined, tool-specific parameters but did not observe significant deviations in terms of performance metrics (data not shown). For similar reasons, we set edit distance to 2 (36/56-bp reads) or 4 (100-bp reads) for read alignment based on the recommendation of 4 % read length (see http://bio-bwa.sourceforge.net/bwa.shtml). Therefore, we only reported in Table 4 the results obtained under default settings for tool-specific parameters, the chosen *k* values (determined by KmerGenie), and the fixed edit distances.

## Conclusions

Identifying and correcting errors in NGS data is an important step before carrying out any further downstream in-depth analysis. This comparative study aimed to provide an independent and unbiased evaluation of the effects of three NGS dataset features on the performance of six recently published *k*-spectrum-based error correction methods with an emphasis on correction accuracy. We observed that performance of six selected methods was dependent on such factors as read length, genome size and coverage depth. Our experimental results suggest that good performance of a method for a specific dataset does not guarantee its ability to perform as well for another type of dataset, hence careful consideration should be given to selecting appropriate tools. Among the six tested methods, Musket appeared to be the front runner, whereas Trowel showed the worst performance. We recommend Musket as the top choice because of its consistently superior performance across all six testing datasets. In future studies, we will expand to other classes of error correction methods and more diversified datasets such as those generated by different NGS platforms (e.g., 454, SOLiD, and Ion Torrent), a wider spectrum of genome size and complexity (e.g., human and mouse genomes), and longer reads (e.g., 300 to 500 bp). More in-depth evaluation is also warranted to investigate other factors like real datasets generated from a wide range of applications (e.g., transcriptome mapping, SNP genotyping, and de novo genome assembly) as well as data structure (e.g., Cucoo filter vs. Bloom filter) on error correction outcomes.
